# Supplementary figures and images for: Identification of Glaesserella parasuis and Differentiation of Its 15 Serovars Using High-Resolution Melting Assays
Source: Pathogens. 2022 Jul 1;11(7):752. doi: 10.3390/pathogens11070752 (PMC9323117; doi:10.3390/pathogens11070752)

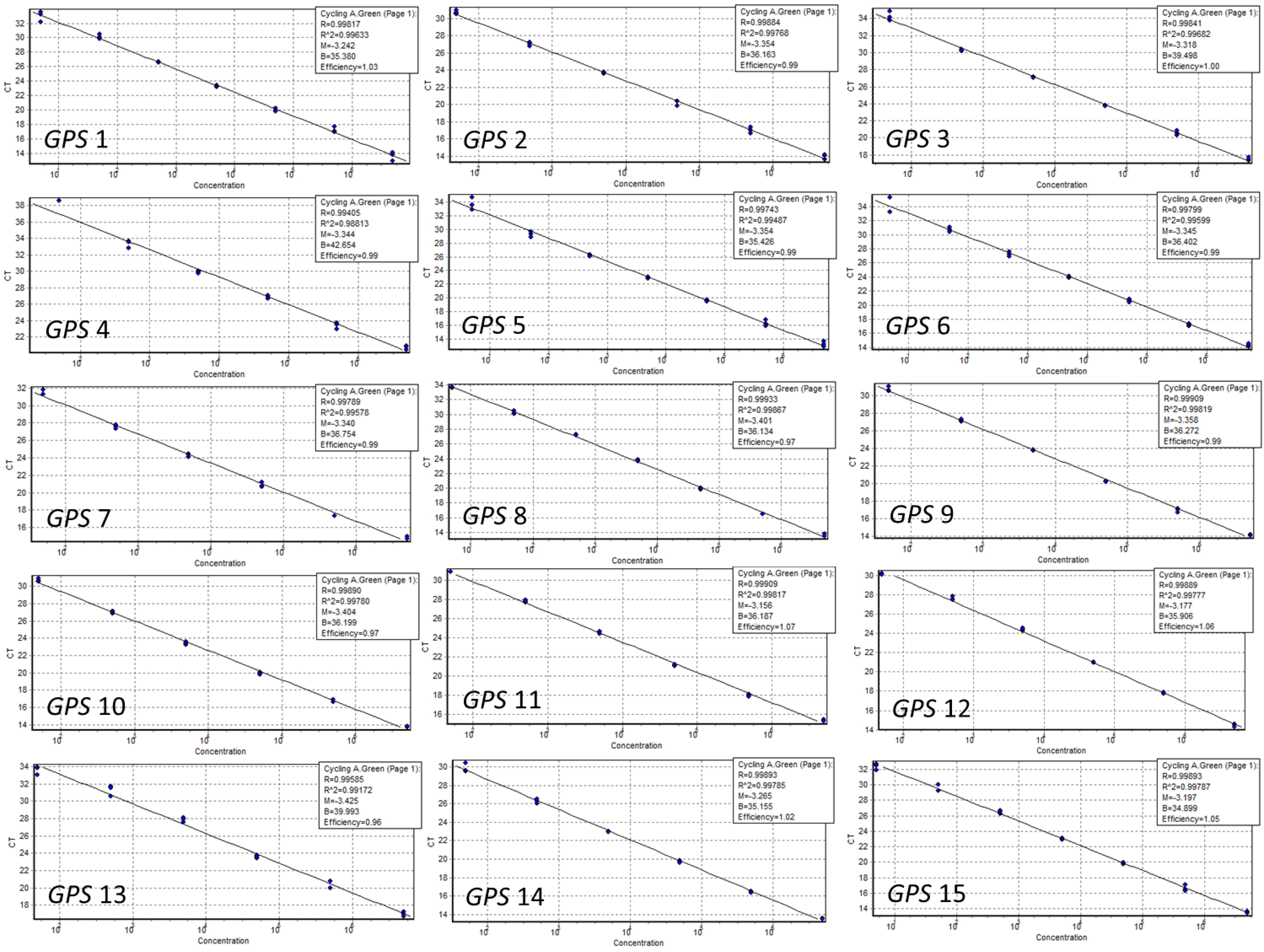

Supplement: Supplementary file 1 [file pathogens-11-00752-s001.zip › Figure S1.tif]

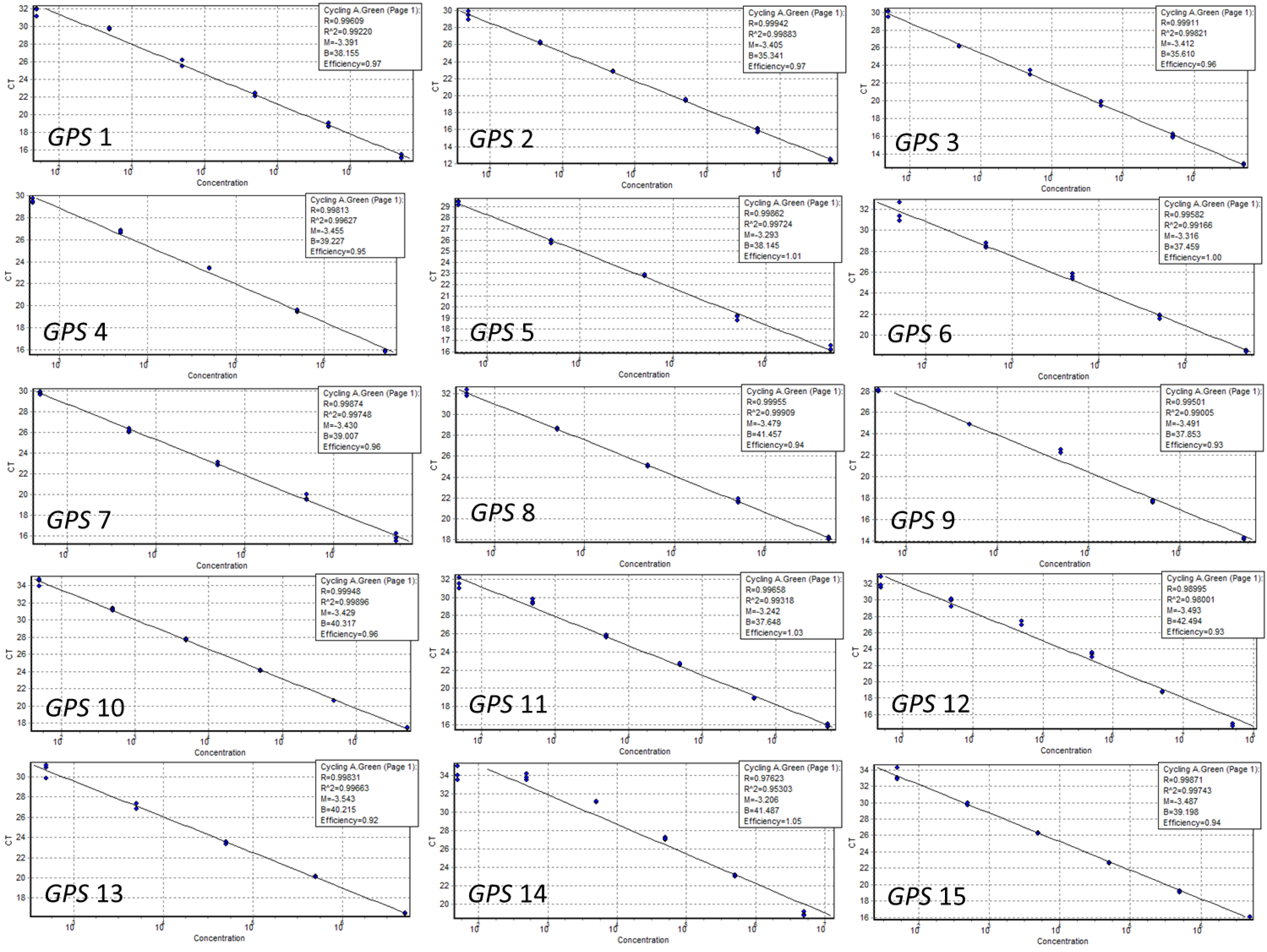

Supplement: Supplementary file 1 [file pathogens-11-00752-s001.zip › Figure S2.tif]

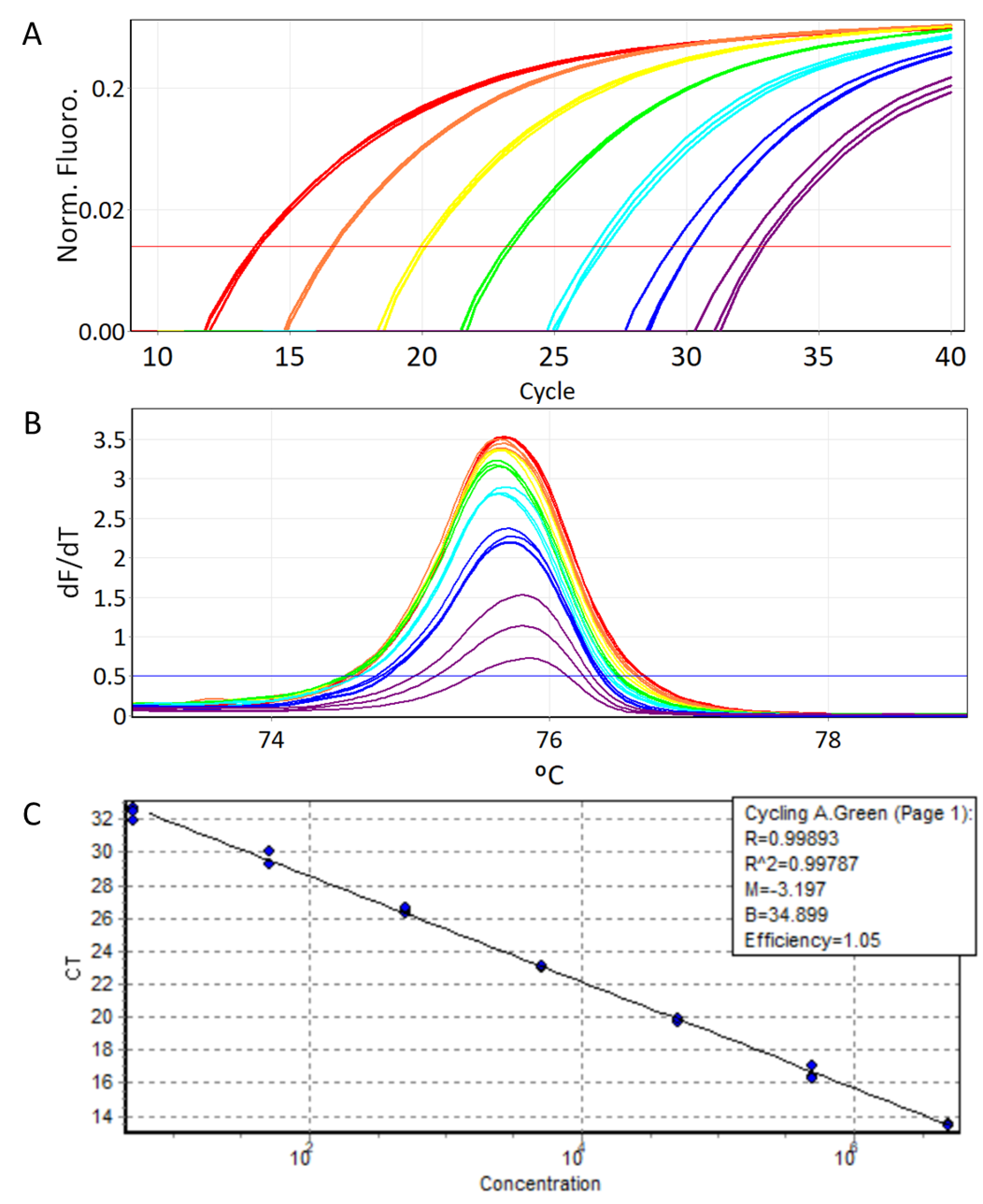

Supplement: Supplementary file 1 [file pathogens-11-00752-s001.zip › Figure S3_NEW.tif]
